# Complexity in Economic and Social Systems

**DOI:** 10.3390/e23020133

**Published:** 2021-01-21

**Authors:** Stanisław Drożdż, Jarosław Kwapień, Paweł Oświęcimka

**Affiliations:** 1 Complex Systems Theory Department, Institute of Nuclear Physics, Polish Academy of Sciences, ul. Radzikowskiego 152, 31-342 Kraków, Poland; jaroslaw.kwapien@ifj.edu.pl (J.K.); pawel.oswiecimka@ifj.edu.pl (P.O.); 2 Faculty of Computer Science and Telecommunication, Cracow University of Technology, ul. Warszawska 24, 31-155 Kraków, Poland; 3 Faculty of Physics, Astronomy and Applied Computer Science, Jagiellonian University, ul. Prof. Stanisława Łojasiewicza 11, 30-348 Kraków, Poland

During recent years we have witnessed a systematic progress in the understanding of complex systems, both in the case of particular systems that are classified into this group and, in general, as regards the phenomenon of complexity [1]. This is possible owing to an outburst of research interest in the science of complexity and a joint effort made by the researchers representing different disciplines and backgrounds which resulted in the enormous number of interdisciplinary studies carried out. This progress has been achieved on both the theoretical, model, and experimental levels. However, in order to comprehend the complexity in full detail, much is still to be done. This is particularly true in the case of the systems involving human society and behaviour.

This Special Issue of *Entropy* was intended to attract researchers specializing in interdisciplinary studies of complex systems, with the economic and social systems in particular, and to collect in one place their contributions that otherwise could be scattered among many journals and issues. We believe that the papers spanning this issue can be considered as valuable input to their specific fields, but also to complexity science in general. Some of them because they relate to general concepts and thus their conclusions can be exploited in various situations across many fields and others because of the methods that were used there, the knowledge of which can be disseminated more broadly. We are glad that our idea was met with a positive response and now we can present as many as 23 genuine research papers on a wide spectrum of topics. The largest set of papers is related to the economical systems, while smaller sets to the social systems and to general complexity, with such a proportion reflecting the total amount of the current scientific output in these fields.

Complexity still lacks a commonly accepted strict definition. In many practical cases it suffices to understand this notion intuitively as a nontrivial, irreducible order (i.e., other than simple regularity or a straightforward effect of a lower level of organization) that spontaneously emerges from an overall chaos [1], but there is also a strong need to provide a strict definition that, for instance, can be applied to categorize various systems based on their structure and dynamics or to construct a measure of complexity. There were a plenty of attempts in that direction but they largely failed. An interesting step towards resolving this issue is presented in a paper [2] where its authors propose a measure of complexity based on a nonlinear transformation of time-dependent entropy that attributes the highest complexity to the optimally mixed states between maximum regularity and maximum disorder.

Various tools based on entropy are frequently used in the context of complex systems and it is not surprising that they are applied in a few other contributed papers. One of the principal directions of research is looking for precursors of the oncoming structural phase transitions. For example, transfer entropy quantifying dependence asymmetry between two systems is used to construct a network of information transfers among cryptocurrencies. The resulting network topology reveals significant alteration during turmoils and forecasts a systemic risk increase [3]. The Tsallis nonextensive entropy has already proved useful in studying complex systems [4]. It is applied to analyze the cross-shareholding networks of companies. In this context it offers a measure of market polarisation and a tool for analyzing market self-organization in response to external shocks [5]. Finally, the moving average cluster entropy is proposed to study the long-range dependence in time series and proves useful as a measure capturing endogenous sources of risk over different temporal horizons [6].

Risk, which quantifies market or asset stability and vulnerability to external shocks, has always been one of the key topics in economics, but it is also an important issue from the complexity perspective [7]. A few more papers from this Special Issue consider systemic risk as one of their central points. If such a risk is quantified in terms of some framework, it is possible to observe its evolution on a given market. For example, the Chinese stock market network topology analysis leads to a conclusion that the systemic risk can be decomposed into a clear trend and periodic fluctuations with the former reflecting the gradual improvement of the management and operation of the market and the latter reflecting the events of excessive strength [8]. Among the most important sources of risk is leverage trading but this relation can rather be non-trivial with either stabilizing or destabilizing impact depending on the leverage trading share in total market activity [9]. In order to manage risk, one needs to construct realistic models that are able to predict the probability of financial losses [10] and to use reliable measures able to provide one with sufficiently early alerts [3]. On the other hand, risk can also be managed by identifying the key companies or sectors that are its sources of centers [11].

Financial markets are among the most interesting complex systems from a perspective of the empirical data analysis, because they provide incomparably clean data. This is why much effort dedicated to studying these markets can be fruitful far beyond the field of economics [1]. Self-organization of the stock markets and their hierarchical structure can be approached from the angle of information transfer between different sectors in various time intervals [12]. This also refers to the cross-shareholding market structure which self-organizes under the influence of external shocks [5]. Both the external shocks and the internal market events can produce excessive demand for information, which, if properly quantified, may offer a way to monitor oncoming market events that are difficult to predict by using other methods. A new tool is proposed based on an internet search engine like the Chinese Baidu [13].

Another signature of market complexity is pricing and timing of the stocks during their initial public offerings. In [14], a few results on the IPO timing properties are considered and discussed. Studying the temporal properties of financial dynamics, which is a property related to their complexity, offers verification for one of the key paradigms of financial markets, namely the efficient market hypothesis [6], and testing for nonlinearity and chaos [15]. Temporal properties of financial dynamics can in turn be one of the consequences of stock liquidity and it is thus important to have a reliable method to quantify it [16].

Same as the most financial markets offer high quality data, the cryptocurrency market offers also a unique possibility of observation of the whole process of new complex system development from scratch [17]. The cryptocurrency market is also interesting as the possible future fate of money. So their evolution and reacting to external shocks like the COVID-19 pandemic is particularly interesting and instructive [3], especially as it seems that this market gradually reaches maturity [18].

Modelling of the economical systems can go beyond the financial markets and also be applied to more general problems like the wealth condensation in society (simple but effective an agent-based model in [19]), the innovation-related performance on a market (the innovation pressure effects of private-owned enterprises and public companies [20]), and the impact of the macroprudential policy on economy and the financial system (with the results on the stabilizing effects of such policy during the turmoils and crises [21]). From this general system level one may look downwards into the system component parts, which reveal complexity on their own: the geographical regions or administrative divisions. It is possible to quantify their economical development by a newly proposed method of the public administration website quality assessment [22] and to analyze differences in an inter-regional business ecosystem structure or economic activity efficiency level by means of a network approach [23].

Complex phenomena occurring on the interface between economical activity and spatial structure are the subject of a study of land speculation on the outskirts of a sample city in Ethiopia [24]. This study investigates motivations the speculators are driven by and concludes on a possible direction local governments should proceed in order to diminish negative impact of such practices on city development. An even more important social phenomenon with a negative impact on the society is fake news. A model of rumour spreading with evolutionary information search dynamics allows one for analyzing optimal search strategies that maximize pay-off for the society and potentially provides the policy makers with the recommendations how to minimize the harmful impact of fake news [25]. The most sociologically-oriented study of this Special Issue considers the research output of the male and female scientists quantified in terms of their publication citations from the perspective of the gender productivity gap [26]. It occurs that a larger gender inequality can be found in the STEM disciplines (i.e., science, technology, engineering, and mathematics) as compared with the non-STEM ones.

Finally, it is worth to mention a more history-oriented essay on the impact of physics (with thermodynamics in particular) on the development of ideas in the contemporary economics [27]. It is an interesting example of the innovation-generating potential of the interdisciplinary cooperation in science, which is the exceptionally welcome in the science of complex systems.

After this introduction, readers are warmly invited to read the papers collected in this Special Issue.
